# Supplementary material for: Non-perturbative terahertz high-harmonic generation in the three-dimensional Dirac semimetal Cd3As2
Source: Nat Commun. 2020 May 15;11:2451. doi: 10.1038/s41467-020-16133-8 (PMC7229177; doi:10.1038/s41467-020-16133-8)
Supplement: Supplementary file 1 — Supplementary Information [file 41467_2020_16133_MOESM1_ESM.pdf]

Supplementary Information

Experimental setup. We performed terahertz (THz) high-harmonic generation (HHG) experiments with THz sources based on a femtosecond laser system and on a linear electron accelerator. For the former, broadband THz radiation was generated through tilted pulse front scheme utilizing lithium niobate crystal. With initial laser pulse energy around 1.5 mJ at 800 nm central wavelength and 100 fs pulse duration broadband THz radiation with up to 3 μJ pulse energy was generated. The spectral distribution of the THz pulses had maximum around 700 GHz. To produce narrow band radiation two bandpass filters (BP1) with central frequency of 670 GHz and 20% bandwidth were applied in the THz beam path (see Extended Data Fig. 1). With parabolic mirrors (OAP) THz radiation was focused onto the sample with spot size of around 500 μm in diameter (FWHM) and 200 nJ pulse energy. To perform electro-optical sampling, 5% of initial laser pulse was split for probe. Wire grid polarizer (WG3) was inserted into the THz beam after the Cd_3As_2 (CdAs) sample for polarization-dependent detection of HHG.

At the linear accelerator in Helmholtz Zentrum Dresden-Rossendorf, multi-cycle superradiant THz pulses were generated in an undulator from ultra-short relativistic electron bunches. The generated THz radiation is carrier envelope phase stable, linear polarized with tunable emitted radiation frequency. Two bandpass filters (BP1) with 300 GHz central frequency and 20% bandwidth were used to substantially suppress radiations other than of 300 GHz. The accelerator was operated at 100 kHz and was synchronized with an external femtosecond laser system. The latter served as probe in electro-optical sampling. To achieve high level of synchronization, pulse-resolved detection scheme was employed. The laser repetition rate was 200 kHz to enable active background subtraction. THz radiation was focused on the Cd_3As_2 sample with the typical spot size of 0.6 mm (FWHM) with 300 nJ pulse energy. For both experiments, THz radiations after the sample was bandpass filtered (BP2) and refocused on to ZnTe crystal for standard electro-optical sampling (see Supplementary Fig. 1). As thickness of the thin-film samples is smaller than the THz wavelength by more than three orders of magnitude, the electric-field strength within the sample was treated as uniform with the value being the average over the sample thickness.

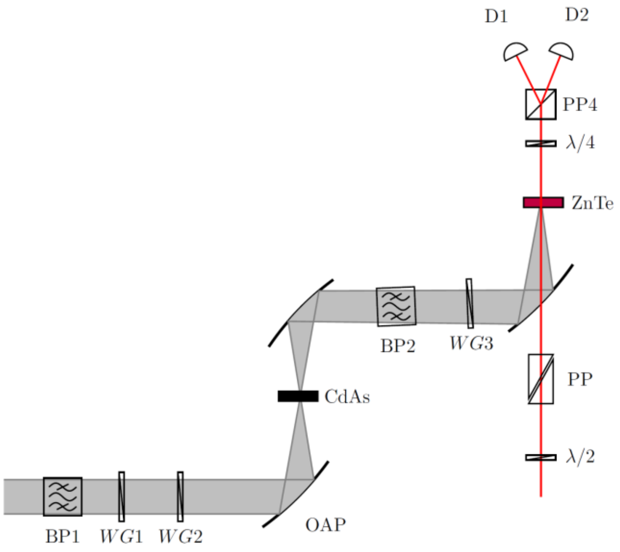

Supplementary Fig. 1 | Sketch of basic experimental setup for high-harmonic generation measurements. BP, band-pass filter; WG, wire-grid polarizer; OAP, off-axis parabolic mirror; $\lambda/2$, half-wave plate; $\lambda/4$, quarter-wave plate; PP, Glan-Tailor prism; PP4, Wollaston prism; D1, D2, photodiode detectors.

Polarization dependence of harmonic radiation. Third-harmonic radiation (THG) of the parallel polarization (with central frequency of 3f = 2.02 THz) were recorded as a function of the pump-pulse polarization corresponding to f = 0.67 THz, as shown in Supplementary Fig. 2. The intensity of the harmonic radiation is nearly independent on the polarization of the pump pulse within the sample surface.

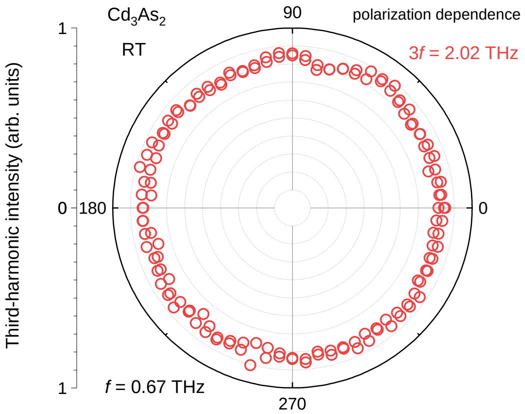

Supplementary Fig. 2 | Polarization dependence of THG. Intensity of third harmonic radiation (3f = 2.02 THz) as a function of pump-pulse polarization at room temperature (RT). The noise level is smaller than the symbol size.

Dependence of harmonic radiation on fluence, scattering rate, and Fermi energy. Different relaxation times are studied theoretically for the pump pulse of f = 0.67 THz as a function of the pump-pulse fluence. The obtained third harmonic intensity is compared for $\tau = 10$ and 30 fs in Supplementary Fig. 3 with the experimental parameters $v_F = 7.8 \times 10^5$ m/s and $E_\mathrm{F} = 118$ meV. The HHG efficiency is very sensitive to the scattering rate. When the scattering rate is reduced by a factor of three, the HHG efficiency can be enhanced up to two orders of magnitude. For the electric-field peak strength of 110 kV/cm, the corresponding current density, time derivative of the current density, as well as HHG spectrum are shown in Fig. 2b, Supplementary Fig. 4a, and Supplementary Fig. 4b, respectively. For $\tau = 10$ fs, not only the third-harmonic, but also the fifth-harmonic radiations should be resolvable. For $\tau = 30$ fs, the efficiency is highly enhanced that harmonic radiations are expected to be detectable at higher orders.

The dependence on scattering rate and electric field is further illustrated by comparing the transient distribution functions. For the electric fields marked in Fig. 2, the distribution functions are illustrated in Supplementary Fig. 5. At the same field strength, the distribution function for $\tau = 30$ fs is much more stretched compared with that for $\tau = 10$ fs, which leads to the much higher HHG efficiency, as shown in Supplementary Fig. 3 and Supplementary Fig. 4.

Based on the kinetic theory, we perform theoretical analysis of the effects of varying Fermi energy, although we cannot freely modify the Fermi energy in the present experiment. As shown in Supplementary Fig. 6, the third harmonic generation increases monotonically with increasing Fermi energy. The waveform of the 0.67 THz pump pulse (see Fig. 2) with a peak field of 56 kV/cm has been used for these simulations.

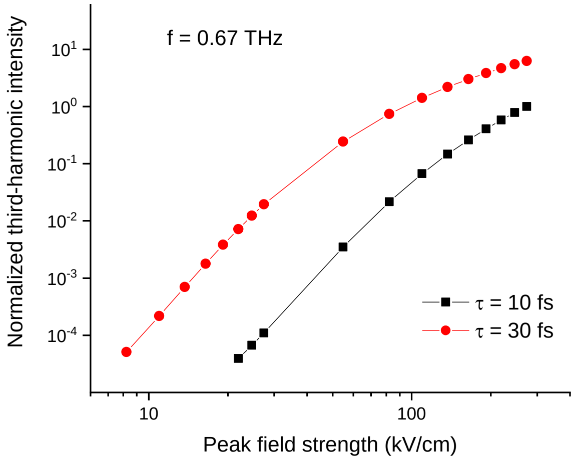

Supplementary Fig. 3 | Theoretical fluence dependence of THG. Normalized intensity of third harmonic radiation (3f = 2.01 THz) as a function of pump-pulse peak field strength for the pump pulse of f = 0.67 THz, obtained by solving the Boltzmann equation.

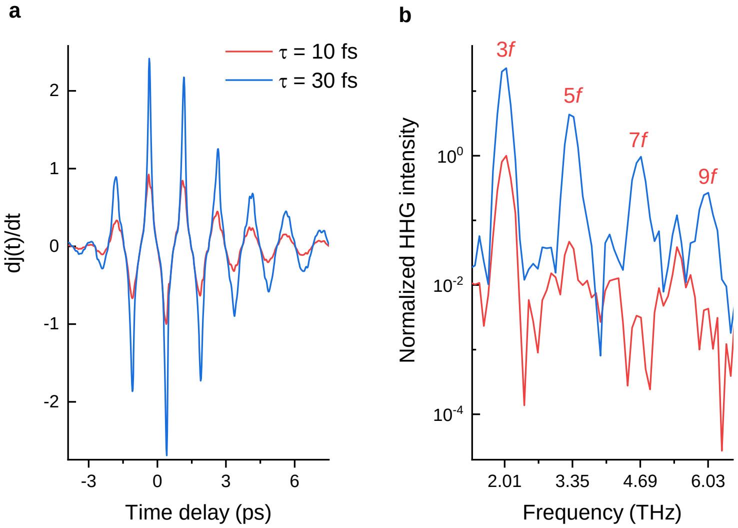

Supplementary Fig. 4 | Theoretical current density and HHG. **a**, Normalized time-derivative of current density $dj(t)/dt$, and **b**, HHG intensity induced by the pump pulse of f = 0.67 THz for $\tau = 10$ and 30 fs, obtained by solving the Boltzmann equation. See Fig.2 for the corresponding current density.

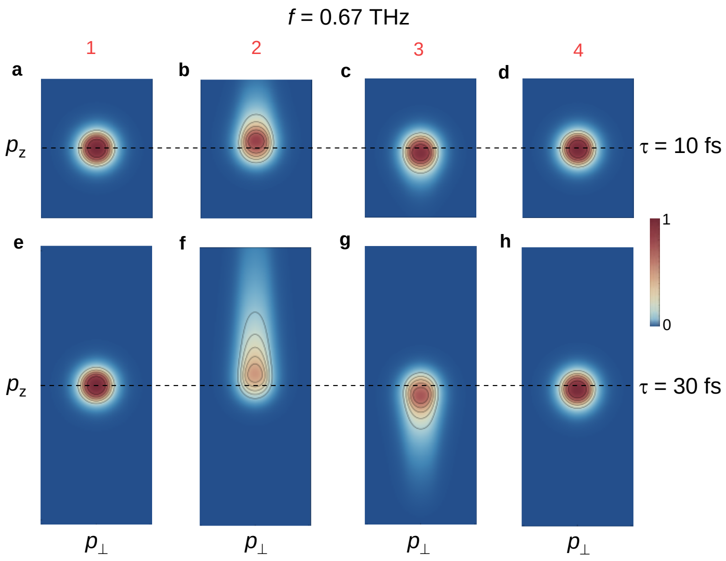

Supplementary Fig. 5 | Theoretical transient distribution function. 2D plots of the transient distribution functions corresponding to different electric fields (marked in Fig.2) for $\tau = 10$ and 30 fs.

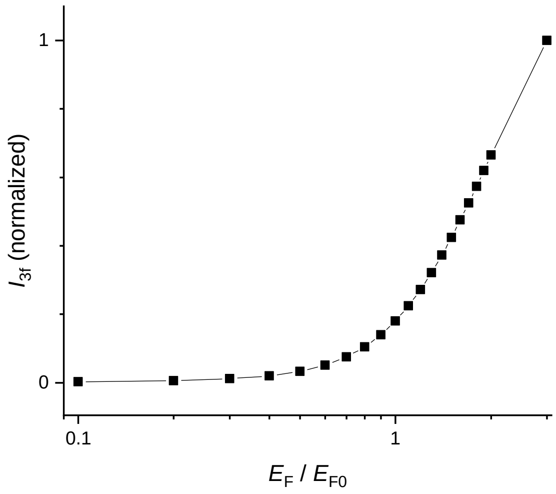

Supplementary Fig. 6 | Dependence of THG intensity on Fermi energy from theory. Normalized THG intensity I_{3f} increases with increasing Fermi energy E_F. The waveform of the 0.67 THz pump pulse (see Fig. 2) with a peak field of 56 kV/cm is used for the simulations. The other parameters are fixed to $E_{F0} = 118\text{meV}$, $v_F = 10^6$ m/s, $\tau = 10$ fs.
