# Supplementary material for: Non-perturbative terahertz high-harmonic generation in the three-dimensional Dirac semimetal Cd3As2
Source: Nat Commun. 2020 May 15;11:2451. doi: 10.1038/s41467-020-16133-8 (PMC7229177; doi:10.1038/s41467-020-16133-8)
Supplement: Supplementary file 3 — Description of Additional Supplementary Files [file 41467_2020_16133_MOESM3_ESM.pdf]

Description of Additional Supplementary Files

File Name: Supplementary Data 1
Description: Distribution function corresponding to τ = 10 fs, for driving pulse of 0.7 THz with peak field of 110 kV/cm

File Name: Supplementary Data 2
Description: Distribution function corresponding to τ = 30 fs, for driving pulse of 0.7 THz with peak field of 110 kV/cm
