# Supplementary figures and images for: Non-perturbative terahertz high-harmonic generation in the three-dimensional Dirac semimetal Cd3As2
Source: Nat Commun. 2020 May 15;11:2451. doi: 10.1038/s41467-020-16133-8 (PMC7229177; doi:10.1038/s41467-020-16133-8)

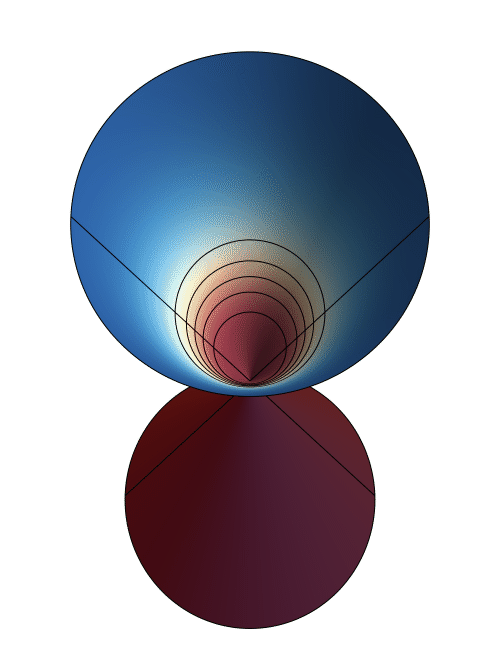

Supplement: Supplementary file 4 — Supplementary Movie 1 [file 41467_2020_16133_MOESM4_ESM.gif]

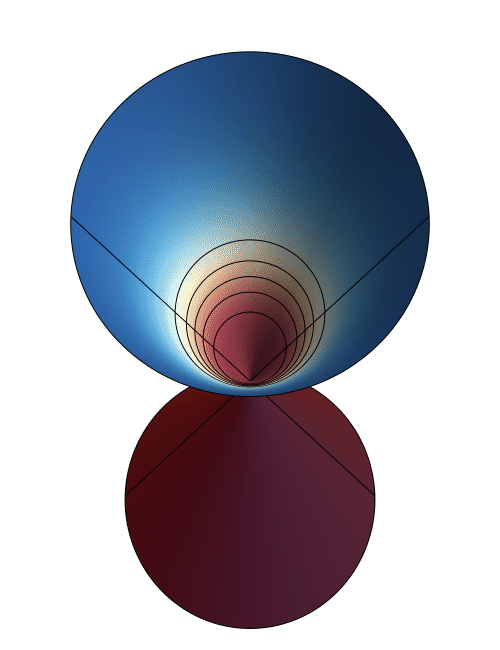

Supplement: Supplementary file 5 — Supplementary Movie 2 [file 41467_2020_16133_MOESM5_ESM.gif]
